# Supplementary material for: A Non-Invasive Neonatal Signature Predicts Later Development of Atopic Diseases
Source: J Clin Med. 2022 May 12;11(10):2749. doi: 10.3390/jcm11102749 (PMC9143112; doi:10.3390/jcm11102749)
Supplement: Supplementary file 1 [file jcm-11-02749-s001.zip › jcm-1666171-supplementary.pdf]

SUPPLEMENTARY TABLES

Supplementary Table S1. Comparison of biomarkers and CT of Archaea according to the presence or absence of maternal antibiotic therapy and according to the mode of delivery.

Parameters	Peripartum maternal antibiotic therapy	p-value	Mode of delivery	p-value
**CT Archaea**	Yes	0.69	Cesarean section	0.90
	No		Vaginal delivery	
**IgE**	Yes	1	Cesarean section	0.98
	No		Vaginal delivery	
**Tryptase**	Yes	0.43	Cesarean section	0.48
	No		Vaginal delivery	
**Calprotectin**	Yes	0.70	Cesarean section	0.07
	No		Vaginal delivery	
**EDN**	Yes	0.83	Cesarean section	0.11
	No		Vaginal delivery	
**TGF-β**	Yes	0.10	Cesarean section	0.40
	No		Vaginal delivery	
**IL-1β**	Yes	0.06	Cesarean section	0.61
	No		Vaginal delivery	
**IL-10**	Yes	0.004	Cesarean section	0.16
	No		Vaginal delivery	
**IL-6**	Yes	071	Cesarean section	0.71

Supplementary Table S2. Comparison of median CT of Archaea in preterm infants according to the presence or absence of allergic events during the first year of life.

Periods	CT Archaea		p-value
	Allergic events		
	Yes	No	
**Meconium**	37.7	36.3	0.98
**Two-weeks**	36.2	37.2	0.28
**Four-weeks**	37.9	37.8	0.88
**Six-week**	38.3	38.2	0.69

Supplementary Table S3. Comparison of median CTs of Archaea in preterm infants according to the presence or absence of atopic dermatitis during the first year of life.

Periods	CT Archaea		p-value
	Atopic dermatitis		
	Yes	No	
**Meconium**	36.3	36.7	0.66

**Two-weeks**	37.2	37.1	0.89
**Four-weeks**	37.9	37.7	0.96
**Six-week**	38.2	38.3	0.83
